# Physician workforce imbalances in Japan: how structural incentives shape hospital-based acute-care capacity

**DOI:** 10.1186/s12960-026-01099-3

**Published:** 2026-09-02

**Authors:** George Kujo

**Affiliations:** Independent Researcher, Tokyo, Japan

**Keywords:** Physician workforce, Japan, Workforce distribution, Physician retention, Working hours, Cosmetic medicine, Gender, Health policy

## Abstract

Japan's physician workforce problem in hospital-based high-intensity care is better understood as an interaction between distribution and retention than as a simple question of national physician headcount. This Comment examines three pathways that can reduce hospital service capacity: working conditions that impede entry and career continuity in high-intensity specialties; early movement from initial residency into cosmetic medicine, known as choku-bi; and later movement from hospital employment to clinic practice. Recent national data show substantial improvement in physician working hours after the 2024 Work Style Reform, but long hours remain concentrated in surgery, emergency medicine, and obstetrics and gynaecology. The national prevalence of choku-bi is not known; professional-society data instead provide a limited indicator of early entry into cosmetic practice. Published income data also show a large difference between salaried hospital practice and clinic ownership, but do not establish that income causes physicians to leave hospitals. These distinctions matter because national supply, specialty distribution, and movement across care settings are analytically different problems. Policy should therefore focus on measurable retention and distribution mechanisms: phasing out routine dependence on the highest service-related overtime ceilings, testing targeted retention incentives for high-intensity hospital functions, strengthening training and competency governance in cosmetic medicine, supporting career continuity, and collecting longitudinal data on physician transitions. Japan illustrates a broader workforce principle: adequate national headcount does not guarantee adequate service capacity when clinicians are difficult to distribute and retain in the settings where they are needed.

## Background

Japan’s hospital physician workforce problem should not be framed as a choice between absolute shortage and maldistribution. National physician numbers have continued to rise, but physicians remain unevenly distributed across regions, specialties, and care settings. The practical outcome can therefore be shortage within particular hospital services even when the aggregate physician stock is increasing. This Comment focuses on hospital-based high-intensity care, defined here as practice involving substantial emergency, operative, unscheduled inpatient, night, or on-call responsibilities. The central question is how working conditions, career structures, and economic incentives shape entry into and retention within these roles.

Hospital working conditions are central to this problem, although recent reforms have produced measurable improvement. In a national 2025 survey of 13 205 full-time hospital physicians, the proportion working 60 h or more per week had fallen substantially compared with earlier surveys. Long hours nevertheless remained concentrated in high-intensity fields: 25.1% of surgeons, 23.7% of emergency physicians, and 22.8% of obstetricians and gynaecologists worked at least 60 h per week [1]. University-hospital physicians also worked longer hours on average than physicians at other hospitals, 52.2 versus 44.3 h per week [1]. Official working-time measures include work at secondary workplaces but exclude some on-call waiting time and, under specified conditions, waiting time during approved night or holiday duty [1]. These data suggest improvement without eliminating substantial specialty and institutional variation.

The 2024 Work Style Reform introduced a general annual overtime ceiling of 960 h for physicians, while designated institutions may use ceilings of up to 1860 h for specified regional-service or training functions [1]. These figures are ceilings, not required amounts of overtime. The government has set a target of ending the regional-service B and linked-B categories by the end of fiscal 2035 [1]. Workforce policy must therefore address not only how many physicians are trained, but also whether hospitals can deliver essential services without persistent dependence on unusually long physician working hours.

## Gendered barriers to participation and retention in high-intensity hospital specialties

The medical-school admissions scandal that became public in 2018 exposed discriminatory practices against female applicants and prompted a national investigation [2]. Any assessment of subsequent change requires attention to training lag. Undergraduate medical education in Japan lasts six years, followed by a mandatory two-year initial residency before most specialty training. Physicians entering medical school after the 2018 scandal therefore had not reached established specialist practice by 2022. The pronounced underrepresentation of women in several surgical specialties in the 2022 national physician statistics should consequently be interpreted primarily as a legacy of earlier training and career structures, not as evidence that post-2018 admissions reforms have failed [3].

Career continuity is a related but distinct issue. In a survey of 711 female graduates from two Japanese medical schools, 55% had left full-time employment at least once; among those who had resigned, 33% had returned to full-time work [4]. Difficulty balancing work with childbirth and child-rearing was a leading reason for resignation, and long working hours were also reported [4]. These findings do not establish a single national return-to-work rate, and they do not justify attributing career interruption to childcare availability or affordability alone. They do support a narrower point: caregiving responsibilities can interact with inflexible and high-intensity working patterns in ways that impair continuity in hospital careers.

Training structure also matters. Analysis of Japan’s 2004 postgraduate residency reform found that female physicians became more likely to enter general surgery and urology after mandatory rotations were introduced, with shifts within surgical fields particularly evident in breast surgery and urology [5]. The study does not show that all surgical fields have comparable working conditions, nor should all surgery be treated as synonymous with acute care. Rather, it suggests that exposure during training and the organisation of work can both influence specialty choice. For workforce planning, the relevant question is whether high-intensity hospital careers can accommodate physicians whose lives include caregiving responsibilities, regardless of gender.

## The choku-bi phenomenon: an early-career exit pathway

A distinct early-career pathway has emerged alongside longer-standing hospital-to-clinic movement. The colloquial term choku-bi (直美) refers to physicians who enter cosmetic medicine directly after completing Japan's mandatory two-year initial residency, without first completing relevant specialty training. Materials submitted by the Japan Society of Aesthetic Surgery (JSAS) to a Ministry of Health, Labour and Welfare (MHLW) advisory panel define choku-bi as entry into cosmetic medicine immediately after residency, within three years of medical licensure [6].

Its national scale is uncertain. Routine national statistics do not identify how many physicians enter cosmetic medicine directly after residency. A more direct, but narrower, indicator comes from JSAS membership data. Across membership-entry assessments from September 2022 to April 2024, physicians joining the society within three years of medical licensure accounted for an average of 32.5% of new entrants, with a range of 18.8% to 46.2% [6]. This denominator is new JSAS entrants, not all residency graduates or all cosmetic practitioners, and time to society membership is not identical to time to employment. The figure therefore cannot be interpreted as a national choku-bi prevalence.

The significance of choku-bi is consequently not that it has been shown to account for a large share of Japan's hospital workforce shortage. Rather, it demonstrates the existence of a pathway through which physicians can leave the hospital specialty-training pipeline immediately after initial residency. JSAS submissions to the MHLW also describe concerns about physicians entering cosmetic practice without sufficient experience in plastic surgery, surgery, dermatology, perioperative management, or emergency management of complications [6]. The aggregate workforce effect remains unquantified, but the pathway is relevant to both workforce distribution and competency governance.

## The hospital-to-clinic pathway and economic incentives

Early movement into cosmetic practice sits alongside a longer-established transition from hospital employment to clinic practice later in physicians' careers. Financial incentives may contribute to that transition. Published data cited in a review of Japanese primary care reported average annual income of approximately ¥14.7 million for hospital physicians in 2017 and ¥24.6 million for physicians running clinics [7]. These figures document an economic differential associated with practice setting and ownership, but they do not establish that income causes physicians to leave hospitals. They also do not capture all differences in working hours, business risk, capital investment, or clinical responsibilities.

Clinic practice should not be treated as a loss to the health system. Clinics provide essential primary and specialist outpatient care, and outpatient practice may preserve substantial specialist expertise. The narrower workforce issue is service capacity. When a physician moves from a hospital role involving inpatient, emergency, operative, night, or on-call responsibilities to a role without those functions, the hospital loses access to that component of service capacity. Whether such transitions are driven mainly by income, working conditions, autonomy, age, family circumstances, or other factors cannot be determined from the available aggregate data.

## Regulatory governance and specialty-entry requirements

The choku-bi pathway is facilitated by the separation between general medical licensure and specialist certification. In Japan, a licensed physician who has completed initial residency can enter cosmetic practice without first completing specialist training in plastic surgery or dermatology [6]. This is not a regulatory exception unique to cosmetic medicine, and the absence of a mandatory specialty-certification gateway is not unique to Japan. In California, for example, the Medical Board states that board certification is not required for a physician to practise medicine, although rules govern how specialty credentials may be advertised [8]. Requirements elsewhere in the United States vary by state.

Japan's policy relevance therefore does not rest on claiming that its framework is uniquely permissive. Instead, choku-bi illustrates a broader governance issue that can arise when licensure, specialist training, and workforce planning are only loosely connected. The appropriate policy response should distinguish competency from workforce protectionism. The strongest immediate case is for transparent reporting of training history, risk-based competency requirements for invasive procedures, and structured supervision or training for physicians without relevant specialist experience. Whether stronger entry restrictions are justified should depend on evidence about patient safety and workforce effects rather than on specialty title alone.

## Historical supply policy and recent distribution reform

Physician training capacity provides important context but should not be treated as a sufficient explanation for present hospital shortages. Medical-school admission capacity expanded from 7625 places in fiscal 2007 to 9403 in fiscal 2024 [9], while national physician statistics show that the total physician stock continued to increase through 2022 [3]. This trajectory makes it difficult to characterise the current problem simply as an absolute national shortage. It is more useful to ask how aggregate supply interacts with distribution across regions, specialties, and care settings.

Recent policy increasingly reflects that distinction. From April 2026, physicians planning to open a new no-bed clinic in a prefecture-designated area with a particularly high concentration of outpatient physicians must notify the prefectural government six months in advance and report the functions they intend to provide [10]. Where a proposed clinic does not contribute to locally undersupplied functions, the prefectural governor may issue a request or recommendation. The reform does not prohibit clinic opening. Instead, a clinic that does not comply may receive designation as an insured medical institution for three years or less rather than the usual six years [10]. This is a meaningful change from the historically permissive approach to clinic location, but it regulates the geographic and functional distribution of new outpatient clinics rather than directly preventing physicians from leaving hospital employment. Its effect on hospital-to-clinic workforce flows remains to be evaluated.

## Policy implications

Addressing hospital workforce imbalances requires policies directed at retention and distribution, with explicit evaluation rather than assumptions about effect. Table 1 summarises four actionable areas.

**Table 1:** Interlocking workforce mechanisms, evidence limitations and policy levers

Mechanism	Evidence and uncertainty	Policy lever and evaluation
Hospital workload and retention	Long working hours have fallen, but ≥ 60 h/week remains concentrated in surgery, emergency medicine and obstetrics/gynaecology. The 1860 h ceiling is an exception, not a target	Use the FY2035 end-date for service-related B/linked-B exceptions as a restructuring deadline; track overtime, vacancies, turnover and service volume
Career continuity in high-intensity specialties	Historical studies show substantial interruption of full-time careers among female physicians; causes are multiple and national return rates are uncertain	Flexible rostering, structured return pathways and fair distribution of night/on-call duties; track specialty entry and full-/part-time retention after interruption
Early entry into cosmetic medicine	JSAS data show many recent entrants close to licensure, but national choku-bi prevalence and specialty-specific workforce effects are unknown	Report training history; introduce risk-based competency and supervision requirements; monitor post-residency destinations before stronger entry restrictions
Hospital-to-clinic movement	A large historical income differential is documented, but causality and current transition rates are not established	Pilot targeted retention payments for high-intensity hospital functions; evaluate turnover, vacancies, working hours and cost before scale-up

First, Japan should treat the existing plan to end the regional-service B and linked-B overtime exceptions by the end of fiscal 2035 as a concrete workforce-restructuring deadline [1]. Hospitals that rely on these categories should demonstrate year-on-year reductions in overtime while replacing lost physician hours through service consolidation, task-sharing, administrative support, and roster redesign. The immediate policy objective is not to assume that 960 h is an ideal final ceiling, but to end routine dependence on the 1860-h service-delivery exception and move hospital practice toward progressively shorter and more sustainable working weeks.

Second, economic policy should focus on testable hospital-retention interventions rather than on reducing clinic income. Targeted retention payments could be piloted for physicians who provide emergency, operative, night, and on-call functions that are difficult to staff. To avoid merely increasing institutional revenue, such payments should be explicitly linked to the targeted work and evaluated against vacancy rates, turnover, physician working hours, and service availability. The 2017 income comparison provides a rationale for testing economic incentives, not proof that remuneration is the dominant cause of movement [7].

Third, regulation of cosmetic medicine should initially focus on measurable competency and transparency. Mandatory reporting of specialist qualifications and training histories, risk-based competency requirements for invasive procedures, and documented supervision or training for physicians without relevant experience are more proportionate first steps than an immediate blanket specialty-certification requirement. National monitoring should also distinguish direct post-residency entry from movement after partial or completed specialty training. Without those data, neither the size of choku-bi nor its effect on individual hospital specialties can be estimated reliably.

Fourth, retention policies should address career continuity rather than treating gender representation as a pipeline problem alone. Institutional childcare may be useful, but the available evidence does not support childcare availability as a sufficient explanation for career interruption. Hospitals and specialty organisations should combine flexible rostering, predictable scheduling where clinically feasible, structured return pathways, and redistribution of night and on-call duties. Progress should be measured through specialty-training entry, full-time and part-time retention after career interruption, and leadership representation, rather than female physician headcount alone.

Finally, Japan needs longitudinal workforce measurement capable of observing transitions. Existing statistics are strong on headcount and cross-sectional practice setting, but much weaker on movement from residency to specialty training or cosmetic medicine and from hospital employment to clinic ownership. Linking career stage, specialty, practice setting, working hours, and transitions would allow policymakers to distinguish stock problems from distribution and retention problems and to evaluate which interventions actually preserve hospital service capacity.

## Limitations

This Comment synthesises official statistics, policy documents, and selected observational studies; it does not estimate causal effects. Several of the mechanisms discussed are therefore plausible workforce pathways rather than quantified determinants of hospital staffing. The national prevalence of choku-bi is unknown, and JSAS membership data are not population-based. The hospital-clinic income comparison is historical and cannot establish either current differences or causal effects on career movement. Studies of female physicians use selected cohorts and differing definitions of resignation and return, so they should not be read as a national return-to-work rate. International regulatory comparison is illustrative rather than comprehensive, particularly because United States requirements vary by state. Finally, working-time conditions changed materially after the 2024 reform; recent 2025 data show improvement, so descriptions of persistent workload problems should refer to remaining specialty and institutional concentration rather than assume uniformly extreme hours across Japanese hospitals.

## Conclusions

Japan's hospital physician workforce problem cannot be reduced to either national physician numbers or maldistribution alone. Hospital service capacity is shaped by the interaction of aggregate supply, distribution across specialties and care settings, and retention in roles that involve substantial emergency, operative, night, and on-call responsibilities. Recent improvements in working hours show that these conditions are not fixed, but the remaining concentration of long hours in high-intensity fields, incomplete measurement of early-career exits such as choku-bi, and economic differences across practice settings all warrant closer workforce attention.

Policy should therefore be judged by whether it changes measurable workforce flows and service capacity, not by whether it simply increases physician headcount or adds new regulatory categories. A staged reduction in dependence on high overtime ceilings, evaluated retention incentives, proportionate competency governance, support for career continuity, and longitudinal tracking of physician transitions offer a more coherent approach. The broader lesson extends beyond Japan: an adequate national stock of clinicians does not guarantee adequate access to high-intensity hospital services when the workforce is difficult to distribute and retain.

## Data Availability

No datasets were generated or analysed during the current study.

## Abbreviations

JSAS: Japan Society of Aesthetic Surgery
MHLW: Ministry of Health, Labour and Welfare (Japan)
